# Supplementary material for: The Comprehensive Analysis of Motor and Neuropsychiatric Symptoms in Patients with Huntington’s Disease from China: A Cross-Sectional Study
Source: J Clin Med. 2022 Dec 27;12(1):206. doi: 10.3390/jcm12010206 (PMC9821667; doi:10.3390/jcm12010206)
Supplement: Supplementary file 1 [file jcm-12-00206-s001.zip › jcm-2049834-supplementary.pdf]

Supplementary Table S1: The subdomain of UHDRS-TMS scale in 205 HD mutation carriers.

| | eye movement | oropharyngeal | hand movements | rigidity/bradykinesia | dystonia | chorea | gait/balance |
|---|---|---|---|---|---|---|---|
| **Total (205)** | 9.19 (6.45) | 2.44 (1.63) | 9.52 (4.60) | 2.30 (2.14) | 2.10 (3.30) | 10.69 (5.2) | 4.16 (2.49) |
| **Male** | 10.11 (6.33) | 2.67 (1.54) | 10.04 (4.79) | 2.39 (2.16) | 2.46 (3.67)[a] | 11.54 (5.62) | 4.02 (2.46) |
| **Female** | 8.55 (6.47) | 2.28 (1.68) | 9.16 (4.45) | 2.13 (2.14) | 1.84 (3.00)[a] | 10.10 (5.39) | 4.25 (2.51) |
| **Juvenile HD (4)** | 11.75 (8.06) | 1.50 (1.73) | 6.25 (2.63) | 4.25 (3.59) | 2.75 (4.27) | 4.00 (4.08)* | 3.75 (0.96) |
| **Adult HD (193)** | 9.02 (6.41) | 2.44 (1.64) | 9.49 (4.55) | 2.22 (2.13) | 2.12 (3.33) | 10.65 (5.42)* | 4.12 (2.46) |
| **Elderly-onset HD (8)** | 12.00 (6.44) | 2.88 (1.46) | 11.88 (5.89) | 1.75 (1.39) | 1.13 (1.64) | 14.88 (5.22)* | 5.38 (3.42) |
| **Positive family history (162)** | 9.37 (6.55) | 2.48 (1.66) | 9.35 (4.35) | 2.19 (2.20) | 2.13 (3.45) | 10.62 (5.60) | 4.13 (2.41) |
| **Paternal inheritance (86)** | 9.29 (6.47) | 2.51 (1.49) | 9.38 (3.90) | 2.09 (1.91) | 2.41 (3.89) | 10.55 (60) | 4.33 (2.02) |
| **Maternal inheritance (76)** | 9.46 (6.68) | 2.45 (1.85) | 9.30 (4.84) | 2.29 (2.49) | 1.82 (2.87) | 10.70 (5.63) | 3.89 (2.78) |
| **Negative family history (43)** | 8.49 (6.70) | 2.28 (1.52) | 10.16 (5.46) | 2.44 (1.93) | 1.98 (2.69) | 10.95 (5.26) | 4.29 (2.78) |
| **Motor (188)** | 9.20 (6.48) | 2.42 (1.64) | 9.54 (4.63) | 2.22 (2.17) | 2.09 (3.31) | 10.80 (5.56) | 4.13 (2.51) |
| **Cognitive (5)** | 9.40 (8.99) | 2.60 (1.52) | 11.00 (5.24) | 1.4 (0.55) | 2.40 (3.58) | 11.20 (6.98) | 5.20 (3.27) |
| **Psychiatric (12)** | 8.92 (5.13) | 2.67 (1.61) | 8.58 (3.97) | 2.92 (1.98) | 2.08 (3.20) | 8.75 (4.07) | 4.09 (1.58) |

[a], *: statistically significant ($p<0.05$)

Supplementary Table S2: The score of neuropsychiatric scales in 205 HD mutation carriers.

| | MMSE | HAMD | HAMA | BDI |
|---|---|---|---|---|
| **Total (205)** | 22.41 (5.32) | 10.19 (6.68) | 10.25 (5.12) | 6.51 (5.90) |
| **Male** | 23.80 (3.86) | 10.08 (6.15) | 11.29 (5.25) | 6.33 (5.51) |
| **Female** | 21.90 (5.63) | 10.29 (7.15) | 9.44 (5.18) | 6.65 (6.27) |
| **Juvenile HD (4)** | 27.67 (1.16) | 2.5 (0.71) | / | 3.00 (1.41) |
| **Adult HD (193)** | 22.28 (5.35) | 10.28 (6.61) | 9.53 (4.39) | 6.56 (5.95) |
| **Elderly-onset HD (8)** | 23.17 (4.62) | 11.60 (8.11) | 16.5 (6.36) | 7.25 (6.70) |
| **Positive family history (162)** | 22.86 (5.03) | 10.11 (6.79) | 8.85 (3.56) | 6.45 (5.90) |
| **Paternal inheritance (86)** | 23.17 (4.68) | 9.94 (6.47) | 8.00 (3.31) | 6.35 (5.45) |
| **Maternal inheritance (76)** | 22.53 (5.40) | 10.32 (7.72) | 9.38 (3.82) | 6.58 (6.53) |
| **Negative family history (43)** | 20.48 (6.14) | 10.67 (6.16) | 16.33 (7.23) | 6.79 (6.13) |
| **Motor (188)** | 22.45 (5.41) | 10.04 (6.47) | 10.25 (5.31) | 6.41 (5.92) |
| **Cognitive (5)** | 20.50 (5.92) | 7.5 (4.95) | 6.5 (4.95) | 2.5 (0.71) |
| **Psychiatric (12)** | 22.64 (4.06) | 11.91(8.63) | 14 (1.41) | 8.38 (6.16) |

UHDRS: Unified Huntington's Disease Rating Scale; MMSE: Mini-Mental State Examination; HAMD: Hamilton Depression Scale; HAMA: Hamilton anxiety scale; BDI: Beck Depression Inventory.

Supplementary Table S3：The comparison of two studies with large sample size of HD patients in China.

| | Li HL | Cheng YF |
|---|---|---|
| Regions of China | Southeastern | Southwestern |
| Sample size | 242 | 205 |
| Sex(male/female) | 131/111 | 84/121 |
| CAG repeat number | 46.1±7.5 | 45.2±4.5 |
| Age of onset/years | 40.3±11.9 | 41.8±10.0 |
| Initial symptoms of motor signs | 215/242(88.8%) | 188/205(91.71%) |
